# The dual impact of irrelevant visual onsets: Habituation of capture unlocks onset facilitation

**DOI:** 10.3758/s13414-025-03185-5

**Published:** 2025-12-11

**Authors:** Andrea Dissegna, Luca Betteto, Matteo De Tommaso, Massimo Turatto

**Affiliations:** 1 Center for Mind/Brain Sciences, Università di Trento, Rovereto, Italy; 2 Università di Milano Bicocca, Milan, Italy

**Keywords:** Attentional capture, Interference, Warning, Temporal orienting, Alerting mechanisms

## Abstract

Irrelevant peripheral visual onsets have consistently been shown to interfere with target processing, a phenomenon attributed to their ability to divert attention from the target. Here we show that in addition to their detrimental effect on performance, irrelevant visual onsets may also facilitate target discrimination. However, this beneficial effect only emerges once habituation mechanisms have fully abolished onset capture. At a 20% onset rate, onsets produced only interference, with capture habituating across blocks of trials. At 50% and 80% rates, a stronger habituation was observed, and once capture was eliminated, onsets began to facilitate performance, as evidenced by faster response times when onsets were present compared to when they were absent. A further experiment demonstrated that visual onsets facilitate performance by allowing temporal expectation about the target moment of appearance, rather than by a generic alerting effect. These findings demonstrate that irrelevant visual onsets trigger two independent processes in the nervous system, resulting in two opposite effects on performance: interference due to attentional capture and facilitation due to temporal expectation. Our results highlight the flexibility of the attentional system in utilizing the same stimulus representation for different purposes, exogenous orienting with subsequent habituation, and temporal orienting, both of which capitalize on stimulus regularities to optimize processing efficiency.

## Introduction

It is well established that sudden events appearing in the visual field tend to capture attention (Müller & Rabbitt, 1989; Posner & Cohen, 1984; Remington et al., 1992; Yantis & Jonides, 1990). This is particularly true in the case of visual onsets, abrupt luminance changes that typically accompany the appearance, even for a brief moment, of new stimuli in the scene (Jonides & Yantis, 1988; Yantis & Jonides, 1984). A long-lasting debate is whether such capture is purely stimulus driven or is at least partially under top-down control. For example, one position holds that visual onsets, and feature singletons more broadly, capture attention in a bottom-up manner due to their intrinsic saliency (e.g., Theeuwes, 2010). In contrast, an opposing view emphasizes the role of top-down control, proposing that salient irrelevant stimuli, including onsets, capture attention only when participants adopt an attentional set that matches their defining features (Folk et al., 1992). This latter perspective, exemplified by the *contingent capture hypothesis*, predicts that irrelevant visual-onset distractors should capture attention only when participants are set for onsets (see also Gibson & Kelsey, 1998, for a similar position).

While several earlier studies failed to find evidence that onsets capture attention unless participants adopted an active attentional set for onsets (e.g., Folk et al., 1992, 1994; Folk & Remington, 1998; Wu & Remington, 2003), thereby supporting the role of top-down factors in attentional capture (e.g., Adams & Gaspelin, 2024; Bacon & Egeth, 1994), more recent work by Folk and Remington (2015), two of the original proponents of the contingent capture hypothesis, has shown that onsets are in fact special visual events capable of capturing attention in a purely stimulus-driven fashion. Indeed, the authors showed that when onsets were relatively rare (occurring in 20% of the trials), they captured attention even though participants were adopting an attentional set for color. The authors acknowledged that this finding, while in agreement with the orienting reflex hypothesis, and its habituation proposed by Sokolov (2002), challenges the contingent capture hypothesis, given that “…infrequent onsets can capture attention independent of a top-down set for color.” Folk and Remington (2015, p. 1162).

So, while the topic concerning the nature of attentional capture and its control is still under discussion and investigation (Luck et al., 2021), capture by irrelevant visual onsets has been shown to be subject to habituation if these events are encountered repeatedly, as predicted by Sokolov’s habituation theory of the orienting reflex (1963; also see Sokolov et al., 2002). In other words, regardless of the specific nature of the onset interference (purely stimulus-driven or mediated by top-down factors), this typically diminishes over time with onsets repetition. This has been largely documented in tasks where participants must discriminate a target that is occasionally preceded by a peripheral visual onset (Kelley & Yantis, 2009; Turatto, Bonetti, & Pascucci, 2018a, 2018b; Turatto, Bonetti, Pascucci, et al., 2018a, 2018b; Turatto et al., 2019, 2024; Turatto & Pascucci, 2016; Valsecchi & Turatto, 2025). On onset-present trials, response times (RTs) for target discrimination are initially longer compared to onset-absent trials, thus attesting distractor interference due to attention being allocated to the irrelevant visual event. However, as the onset distractor repeats across the different blocks of trials, capture progressively declines, and depending on the specific conditions, this may be attenuated or vanish completely (but see Adams et al., 2023; Ruthruff et al., 2019). Previous onset-distractor studies have shown that this effect is a form of habituation of attention (Turatto, 2023), bearing similarities with the habituation of various responses elicited by irrelevant salient stimuli as observed in both humans and other animals (Thompson, 2009).

Habituation of onset capture is influenced by the temporal and spatial predictability of the distractor, which is indeed faster and/or stronger when the onset occurs at an expected time interval (Turatto & De Tommaso, 2022) or spatial location (Turatto & Valsecchi, 2023; Valsecchi & Turatto, 2023). This pattern reflects the operation of an adaptive filtering mechanism that, as postulated by Sokolov (1960, 1963; see also Sokolov et al., 2002), optimizes information processing by exploiting environmental regularities to attenuate the detrimental impact of irrelevant salient stimuli. Accordingly, habituation of the orienting response occurs when the current sensory input matches the expected one, meaning that habituation results from a prediction-error minimization process, where the “error” reflects the discrepancy between the expected and actual sensory input. As the distractor becomes less and less surprising with experience, its capacity to attract attention, and therefore to interfere with the task at hand, is progressively attenuated (for an explanation of distraction rejection in terms of habituation during visual search tasks, see Won, 2021; Won & Geng, 2020; also see Vatterott & Vecera, 2012, for another example of color singleton capture attenuation based on the distractor color rate). Conversely, if the distractor remains a relatively rare event, habituation can be minimal or even absent, and onsets can continue to elicit a reliable orienting response (Folk & Remington, 2015; Neo & Chua, 2006). This expectation-based habituation mechanism helps explain why temporal (Turatto & De Tommaso, 2022) and spatial (Turatto & Valsecchi, 2023; Valsecchi & Turatto, 2023) regularities facilitate the habituation of attentional capture: the more predictable a distractor is, the smaller the prediction error it generates, and the more easily it is ignored. In other words, the less surprising a stimulus is, the less likely it will capture attention (Itti & Baldi, 2009).

The effects of onset statistical regularities so far have been studied in relation to their possible role in the distractor rejection mechanisms. However, unlike the classic additional-singleton paradigm mainly used to study feature-singleton capture (Theeuwes, 1992), where the target, the non-target elements, and the distractor are presented simultaneously in the same display, onset distractors typically precede the target by a brief interval (about 100–200 ms), thus introducing another important temporal regularity in the pattern of sensory stimulation. The fact that the onset distractors systematically anticipate the target could in principle be exploited by the attentional system to optimally prepare for its occurrence, thus enhancing task performance. Indeed, Posner and Boies (1971) originally showed that when a stimulus regularly anticipates the imperative one, it can help to respond more effectively to the latter. This “warning” effect is generally ascribed to a temporary, heightened state of alertness or arousal which improves the speed and accuracy of responses for a short period after the occurrence of the warning signal. Alternatively, anticipatory signals can serve as temporal cues, allowing individuals to optimally prepare by orienting attention to the specific moment in time when task-relevant information is presented (Denison, 2024). This process also reduces uncertainty regarding the timing of target occurrence, thus speeding up RTs (see Los & Agter, 2005).

Hence, although irrelevant visual onsets have mainly been studied in relation to their capacity to attract attention, and to act as potential distractors, the fact that they systematically anticipate the target could also have a facilitatory effect on the processing of the latter. However, any potential beneficial effects of onsets may be obscured by the strong detrimental impact of the attentional capture they elicit, with facilitation, if any, only becoming evident once habituation has abolished this exogenous response.

In the present study, we sought to address this possibility, namely that onsets may have a dual effect on the visual attention system, eliciting both facilitation and interference, probably tapping onto the alerting and orienting system, respectively (Fan et al., 2002; Posner & Boies, 1971). To this aim, we manipulated the onset rate across three experiments to the point where the temporal contingency between the irrelevant onset and the target would be high enough to lead to a complete disappearance of capture, thus potentially leaving room for the facilitatory effect to emerge. This possible scenario finds preliminary support in the study of Turatto and Pascucci (2016), which found that after attentional capture was entirely abolished on the first day of training for an onset with a 50% rate, RTs for target discrimination were generally faster in onset-present trials than in onset-absent trials during the second and third training days. Although this difference was not statistically significant, it suggested to be a potential facilitatory effect of the irrelevant onset on the target discrimination task. Furthermore, De Tommaso and Turatto (2023) observed a similar pattern within blocks of a single training session with a 60% onset rate (see Fig. 2, p. 2534). However, in this case, the trend was not confirmed by any specific statistical analysis.

To obtain more compelling and decisive evidence, here we tested three groups of participants, where each group had to discriminate the orientation (left vs. right), with respect to the vertical axis, of a Gabor patch (the target) presented at the center of the screen. In some trials, a bright white disk briefly appeared before the target in one of four eccentric positions with equal probability. Across Experiments 1–3, we manipulated the overall rate of the irrelevant peripheral onset, which appeared with a 20%, 50%, or 80% rate, respectively.

On the basis of our previous studies (Turatto & Pascucci, 2016), we predicted that in the 20% rate condition onsets should initially produce the largest capture effect, namely the strongest interference with target discrimination, but such interference should gradually diminish across blocks of trials, consistent with habituation. We further expect capture attenuation to be more pronounced for higher global onset rates (50% and 80%), which could progressively lead to a greater onset facilitation on task performance. Specifically, onset facilitation, if any, should be more likely to become evident at the 80% onset rate than at the 50% onset rate, as habituation should be stronger and emerge earlier in the former case.

Any onset interference or capture was measured as the difference between onset-present and onset-absent trials (present minus absent) in both RTs and error rates. Hence, a positive significant difference with 0 attests onset capture, whereas a negative significant difference would document a facilitatory onset effect on performance.

## Experiment 1

In this first experiment, we aimed to replicate the basic finding reported in previous studies, namely that a relatively infrequent visual onset (20% rate, 5% at each of the four positions) captures attention, even when attention is focused at the cued location (e.g., Turatto & Pascucci, 2016). Such capture is then expected to habituate across the different blocks of trials. However, due to the low onset rate, and based on our previous work, we predict that capture while diminishing could remain significant in the final block of trials, which should prevent any beneficial effects of the onset from becoming visible. If confirmed, these results would form the basis for examining how increasing the onset rate influences target processing, as explored in the following two experiments.

### Method

#### Sample size justification

Since our dependent measure was capture (defined as before), the experimental design included a single within-participants factor, Block (block 1, block 2, block 3, and block 4). Based on previous literature (Turatto & Pascucci, 2016), we expected the onset effect to be positive, thus revealing an interference or capture, which should decrease from block 1 to block 4 consistent with a habituation phenomenon.

Based on our research design, and the existing literature, we expected to observe a moderate effect size for the main effect of Block. We conducted an a priori power analysis (using G*Power, Faul et al., 2007) estimating a η_p_^2^ = 0.06 to determine the sample size. The analysis indicated that 23 participants were sufficient to achieve a power of 80% with α =.05. Drawing from our experience with online experiments, we decided to increase the estimated sample size to 25 individuals to ensure an adequate number of participants with an error rate smaller than 20%.

#### Participants

Participants (N = 25) were recruited online through the Prolific service (Prolific Academic Ltd, Oxford, UK). We required them to be English native speakers, to have normal or corrected-to-normal vision, and to run the experiment on a desktop computer. The limitations concerning language skills were implemented because the instructions were delivered in English through the online server, but we would not assume that language or culture per se would affect the results of our experiment. We included a screening set for age to recruit participants in the range of 18–40 years old. Participants were informed about the general aim of the experiment, their task, and data-handling procedures in the Prolific interface. They gave their consent by agreeing to be directed to the experiment URL and were paid £9/h for their participation. All the experiments in the present study were carried out in accordance with the Declaration of Helsinki.

Participants of this experiment were on average 30 years old (SD = 6). There were 22 males (i.e., 88% of the group).

#### Stimuli and procedure

The experiment was programmed using the PsychoPy3 version 2020.1.3 software (Peirce et al., 2019) and run online using the Pavlovia web hosting service (Open Science Tools Limited, Nottingham, UK). Stimuli dimensions are reported as degrees of visual angle assuming a monitor height of 19.5 cm and a viewing distance of 58 cm.

The trial began with the presentation of a white fixation cross (0.3° × 0.3°) at the center of a black screen. The cross remained visible for a jittered duration, randomly varying between 800 and 1,200 ms, in increments of 50 ms.

In onset-absent trials, the fixation cross was replaced by the onset of a Gabor patch (sinusoidal texture with a Gaussian mask, phase = 0, spatial frequency = 7 cycles/° and a radius of 0.50°) that remained visible for 200 ms. The upper part of the Gabor patch was randomly tilted either clockwise or counterclockwise by 4° from the vertical axis. Onset-present trials were as for the onset-absent trials, except that 100 ms before the Gabor appearance an irrelevant peripheral stimulus consisting of a full-contrast white disk was shown for 100 ms on the screen. The disk had a radius of 1.5° and was located at an eccentricity of 6° from the fixation point, along either the vertical or the horizontal axis, in one of four possible positions: above, below, left, or right.

**Fig. 1:**
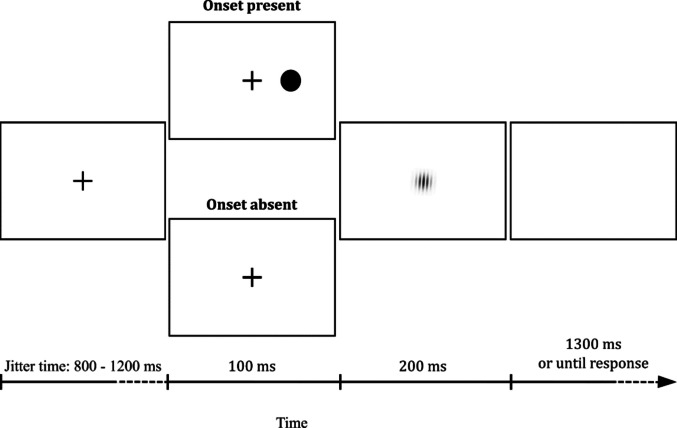
Stimuli and events of Experiment 1. Participants reported the orientation of the Gabor patch (clockwise vs. counterclockwise, with respect to the Gabor upper part) appearing at the center of the screen. In onset-present trials (upper panel), a white disk abruptly appeared for 100 ms in one of four possible positions (above, below, to the left, or to the right of the fixation cross) before the Gabor onset. Stimuli are not drawn to scale

Participants had to discriminate as quickly and accurately as possible the orientation of the Gabor by pressing the right arrow of the keyboard if the Gabor upper part was tilted clockwise or the left arrow if it was tilted counterclockwise. From the target appearance participants had 1,500 ms after to respond, and received feedback for incorrect or absent responses, with the messages “Wrong!” or “Too Slow!” displayed in white letters for 800 ms. The inter-trial interval was set to 1,000 ms, during which a blank screen was displayed. The sequence of events in each trial is shown in Fig. 1.

The experiment began with a practice block of five trials without the onset, followed by four experimental blocks of 100 trials each. In the experimental blocks, onset-present trials were 20% of the total trials, with the disk position chosen at random among the four axes as indicated before.

#### Data analysis

Participants with an error rate > 20% were excluded from the sample. Attentional capture (defined as before) was measured by considering both error rates and RTs and by means of a one-way repeated-measures ANOVA with Block as factor. Before analyzing the effect of onsets, we excluded incorrect responses from the dataset and conducted an outlier analysis on the remaining data to identify and exclude RTs exceeding 2.5 SD above or below the individual mean of each participant.

We reported Greenhouse–Geisser-corrected results whenever Mauchly’s test returned a violation of the sphericity assumption. Post hoc comparisons were conducted using Tukey’s *t-*tests, while planned one-tailed one-sample *t*-tests were used to test, for each level of the factor Block, whether the onset effect, i.e. capture, was greater than 0.

As estimates of the effect size, we provided: (1) partial eta squared (η_p_^2^) for the interactions and main effects of the ANOVA *F*-tests, and (2) Cohen’s *d* for the *t*-tests.

Data were analyzed using R, version 4.2.1 (R Core Team, 2023) and the package dplyr (Wickham et al., 2022), tidyr (Wickham & Maximilian, 2022), ggplot2 (Wickham, 2016), afex (Singmann et al., 2022), and emmeans (Lenth, 2022).

### Results and discussion

All the 25 recruited participants had an error rate < 20% and were included in the analyses. Mean error rates are reported in Table 1. The ANOVA on error rate differences showed no significant effect of Block, *F*(2.41, 57.90) = 1.32, *p* =.276, *ηη*^*2*^_*p*_ =.052.

**Table 1:** Mean error rates and response times (RTs) by Block and Trial type for Experiment 1

**Block**	**Trial type**	**Error rate (SD)**	**RT (SD)**
1	Onset absent	7 (8)	446 (63)
1	Onset present	5 (7)	482 (68)
2	Onset absent	6 (8)	450 (60)
2	Onset present	5 (6)	468 (73)
3	Onset absent	7 (6)	452 (62)
3	Onset present	7 (6)	470 (67)
4	Onset absent	5 (5)	455 (65)
4	Onset present	6 (8)	465 (65)

Mean error rates and their SDs are reported as percentages. Mean RTs and their SDs are reported in milliseconds

**Fig. 2:**
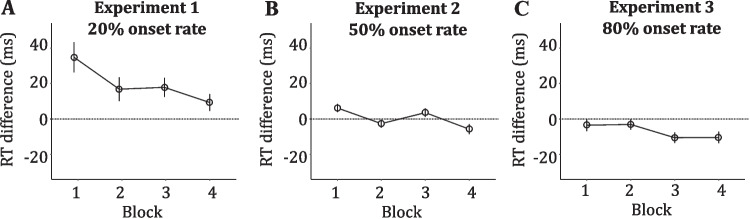
Effects of the onset presence on target discrimination as a function of block and onset rate used in Experiments 1–3. Mean response time (RT) difference (RT onset-present trials – RT onset-absent trials) by onset rate (20% in Experiment 1, 50% in Experiment 2, and 80% in Experiment 3). Bars represent ± 1 standard error of the mean

The outlier-latency criterion resulted in the removal of 2.3% of the correct responses. Absolute mean RTs of correct responses for each condition are reported in Table 1. The ANOVA on the RT differences (Fig. 2, panel A) revealed a significant main effect of Block, *F*(2.59, 62.24) = 5.14, *p* =.005, *η*^*2*^_*p*_ =.176. Post hoc comparisons showed that onset capture was greater in block 1 (*M* = 35, *SD* = 42) than in block 4 (*M* = 9, *SD* = 23), *t*(24) = 3.22, *p* =.017, *d* = 0.660; in addition, capture was significant (compared to 0) in all blocks: block 1, *t*(24) = 4.13, *p* <.001, *d* = 1.690; block 2, *t*(24) = 2.54, *p* =.017, *d* = 1.040; block 3,* t*(24) = 3.41, *p* =.002, *d* = 1.390; block 4, *t*(24) = 2.07, *p* =.048, *d* = 0.850.

The results confirmed previous findings by demonstrating that a relatively infrequent onset (20% rate, 5% in each of the four locations) captured attention, producing a significant increment of RTs during target discrimination compared to onset-absent trials. However, in line with the habituation account onset, interference decreased across blocks, although it also remained significant in the last block of trials (also see Turatto & Pascucci, 2016). According to our hypothesis, the presence of the interference effect in all blocks may have obscured any potential facilitatory effect of onset on target discrimination.

To unveil such a hypothetical dual effect of onsets, in the next experiment the onset rate was increased to 50% of the total trials. This should produce a stronger and faster habituation of onset capture (also see Turatto & Pascucci, 2016), which, in case habituation reduced capture completely, should allow the facilitation effect of onsets on task performance to emerge.

## Experiment 2

Experiment 2 was identical to the previous one, with the exception that the overall onset rate was 50% (12.5% in each of the four locations). Due to the increase rate, which is known to accelerate and strengthen habituation (Turatto & Pascucci, 2016), we expected capture to already be smaller than in Experiment 1 in the first block, and potentially reaching a zero asymptotic level in the following blocks.

### Method

#### Sample size justification

The experimental design included one within-participants factor, Block (block 1, block 2, block 3, and block 4). Based on our research design, the existing literature, and the greater onset rate, we expected to observe a small-to-moderate effect size for the main effect of Block. We conducted an a priori power analysis (using G*Power, Faul et al., 2007) estimating η_p_^2^ = 0.03 to determine the sample size. The analysis indicated that 46 participants were sufficient to achieve a power of 80% with α =.05. We decided to increase the estimated sample size to 48 individuals to ensure an adequate number of participants with an error rate smaller than 20%.

#### Participants

We recruited 48 participants, who had a mean age of 28 years (*SD* = 6). Among them, 21 were female (i.e., 44% of the group). Data for this experiment were collected in 2024.

#### Stimuli and procedure

These were as in Experiment 1, with the exception that the onset rate was 50%.

#### Data analysis

The analytical plan and the software used for the statistical analyses were as in Experiment 1.

### Results and discussion

We excluded one participant whose error rate was > 20%. Mean error rates are reported in Table 2. The ANOVA on error rate differences showed no significant effect of Block, *F*(2.79, 128.31) = 1.67, *p* =.181, *η*^*2*^_*p*_ =.035

**Table 2:** Mean error rates and response times (RTs) by Block and Trial type for Experiment 2

Block	Trial type	Error rate (SD)	RT (SD)
1	Onset absent	5 (4)	484 (68)
1	Onset present	7 (6)	490 (72)
2	Onset absent	5 (5)	484 (68)
2	Onset present	6 (5)	481 (70)
3	Onset absent	5 (4)	479 (66)
3	Onset present	6 (5)	483 (70)
4	Onset absent	7 (6)	480 (65)
4	Onset present	6 (5)	474 (68)

Mean error rates and their SDs are reported as percentages. Mean RTs and their SDs are reported in milliseconds

The outlier-latency criterion resulted in the removal of 2.59% of the correct responses. Absolute mean RTs of correct responses in each condition are reported in Table 2. The ANOVA on the RT differences (Fig. 2, panel B) revealed a significant main effect of Block, *F*(2.80, 128.79) = 6.37, *p* <.001, *η*^*2*^_*p*_ =.122. Post hoc comparisons attested a small but significant onset interference in block 1, (*M* = 6; *SD* = 15), *t*(46) = 2.70, *p =*.009, *d* = 0.40; by contrast, in block 4 the RT difference was negative (*M* = −6; *SD* = 19) and significantly smaller than 0, *t*(46) = −2.02, *p* =.048, *d* = −0.300, thus revealing onset facilitation on target processing.

At a 50% rate, peripheral onsets still captured attention in the first block of trials, although as expected capture was overall smaller than in the same block of Experiment 1 (*p* <.001). Importantly, however, as exposure to onsets continued capture habituated and eventually disappeared, which allowed the onset facilitatory effect to become evident in the fourth block. In other words, the irrelevant white disk that initially acted as distractor later on facilitated participants’ responses in the final block, possibly due to its alerting properties (Posner & Boies, 1971). In the following experiment, we increased the onset rate to 80%, a condition that should strengthen even further the habituation phase, thus possibly anticipating the onset facilitatory effect with respect to the last block.

## Experiment 3

The experiment was identical to the previous one, with the exception that the overall onset rate was 80% (20% in each of the four locations). Because of such a high onset rate, and based on the results of Experiment 2, capture could be negligible or even completely absent starting from the first block; conversely, as compared to Experiment 2, the facilitatory effect of onsets should emerge earlier and/or in a more robust fashion.

### Method

#### Sample size justification

The experimental design was the same as in Experiments 1 and 2. Based on the research design, we estimated a small-to-moderate effect size for the main effect of Block (η_p_^2^ = 0.03). The a priori power analysis indicated that 46 participants were sufficient to achieve a power of 80% with α =.05, but we increased the estimated sample size to 48 individuals to ensure an adequate number of participants with an error rate smaller than 20%.

#### Participants

We recruited 48 participants, who had a mean age of 27 years (*SD* = 6). Among them, 16 were female (i.e., 33% of the group). Data for this experiment were collected in 2024.

#### Stimuli and procedure

These were as in Experiment 1, but the peripheral onset was present in 80% of the trials.

#### Data analysis

The analytical plan and the software used for the statistical analyses were as in Experiment 1.

### Results and discussion

Mean error rates are reported in Table 3. The ANOVA performed on error rate differences revealed no significant effect of Block, *F*(2.86, 134.24) = 1.10, *p* =.349, *η*^*2*^*ₚ* =.023.

**Table 3:** Mean error rates and response times (RTs) by Block and Trial type for Experiment 3

Block	Trial type	Error rate (SD)	RT (SD)
1	Onset absent	6 (7)	487 (71)
1	Onset present	7 (6)	483 (79)
2	Onset absent	5 (6)	480 (58)
2	Onset present	7 (6)	477 (61)
3	Onset absent	5 (6)	490 (63)
3	Onset present	8 (6)	480 (63)
4	Onset absent	8 (9)	491 (68)
4	Onset present	8 (7)	480 (64)

Mean error rates and their SDs are reported as percentages. Mean RTs and their SDs are reported in milliseconds

The outlier-latency criterion resulted in the removal of 2.44% of the correct responses. Absolute mean RTs of correct responses in each condition are reported in Table 3. The ANOVA on the RT differences (Fig. 2, panel C) showed no significant effect of Block, *F*(2.60, 122.23) = 1.91, *p* =.139, *η*^*2*^*ₚ* =.039. However, post hoc comparisons showed that although the onset had a facilitatory effect (i.e., the RT difference was negative) in all blocks, such a difference was statistically significant only in the last two blocks: block 3 (*M* = −10.54; *SD* = 20), *t*(47) = −3.50, *p* =.001, *d* = −1.020; block 4 (*M* = −10.41; *SD* = 22), *t*(47) = −3.21, *p* =.002, *d* = −0.940.

In line with our hypothesis, the presentation of the peripheral onset at a high 80% rate made habituation faster and stronger, as attested by the fact that no onset interference was already found in the first block. This allowed the onset facilitatory effect to emerge earlier (block 3) as compared to Experiment 2 (block 4), and to remain significant till the last block.

### Cross-experiment analysis

To provide stronger support for our main finding regarding the dual role of onsets as a function of their rate of occurrence, we conducted a cross-experiments analysis comparing the average RT differences across blocks in Experiments 1, 2, and 3. To this aim, we conducted a mixed ANOVA on the RT differences with Block (1–4) as within-participants factor, and Onset rate (20%, 50%, and 80%) as between-participants factor. The results revealed a main effect of the factor Block, *F*(2.75, 322.11) = 10.78, *p* <.001, *η*^*2*^*ₚ* =.084, a main effect of the factor Onset rate, *F*(2, 117) = 25.35, *p* <.001, *η*^*2*^*ₚ* =.302, and a Block × Onset rate interaction, *F*(5.51, 322.11) = 2.46, *p* =.028, *η*^*2*^*ₚ* =.040.

To further investigate the interaction, we conducted a post hoc trend analysis to evaluate whether the RT differences across blocks changed in a different manner across the experiments. The results showed a significant negative linear trend in the RT differences for the 20% onset rate, *b* = –75.32, *SE* = 20.17, *t(117*) = –3.73, *p* <.001, *d* = 0.69, indicating that the RT differences decreased steadily in each successive block. By contrast, neither the 50%,* b* = –29.60, *SE* = 14.71, *t(117*) = –2.13, *p* =.132, *d* = 0.37, nor the 80%, *b* = –28.22, *SE* = 14.56, *t(117*) = –1.93, *p* =.155, *d* = 0.36, onset-rate conditions revealed any significant trend.

The lack of negative decrements in the RT differences across blocks in the 50% and 80% onset-rate conditions should not be confounded with a lack of habituation in these experiments. This is because the higher the distractor rate the faster the attentional capture is extinguished, potentially leading to a complete habituation (i.e., capture approaching an asymptotic level) within the initial block of trials. Indeed, it should be noted, that in the 80% rate condition after the first ten trials participants were already exposed, on average, to eight distractors, which may have boosted habituation of capture. However, to address whether capture was present at the beginning of block 1 in each experiment and then decreased as a function of the onset rate, we implemented a bin analysis on the cumulative RT differences. Specifically, for each condition we computed the RT differences across the first *N* trials of block 1, with *N* that was a multiple of five trials. We opted for a minimal five-trial bin width as this value was the smallest bin that contained enough onset-present trial in the 20% condition to conduct a reliable statistical analysis (i.e., this bin width contained at least one onset-present trial per participant). We then performed one-sample *t*-tests against zero on each bin-width to determine whether capture was significant and then ran between-participants ANOVAs to compare the capture magnitude across the 20%, 50%, and 80% experiments at each bin width. This approach allowed us to track down how quickly capture diverged over trials in block 1 as a function of onset rate.

When the first five-trial bin was considered, capture was significantly greater than zero in all conditions (20%: *M* = 59, *SD* = 60, *t*(97) = 4.92, *p* <.001; 50%: *M* = 25, *SD* = 61, *t*(97) = 2.84, *p* =.005; 80%: *M* = 28, *SD* = 77, *t*(97) = 2.57, *p* =.016), with the difference across conditions that only approached significance, *F*(2, 97) = 2.80, *p* =.060, η^2^ₚ =.055. Nonetheless, at the numerical level, capture for the 50% and 80% conditions was already smaller than for the 20% condition. At the larger ten-trial bin, instead, the capture functions significantly diverged, *F*(2, 111) = 3.99, *p* =.021, η^2^ₚ =.067. Capture was still significant in the 20% and 50% conditions, *t*(111) = 4.09, *p* <.001, and *t*(111) = 3.05, *p* =.002 respectively, but disappeared in the 80% condition, *t*(111) = 0.71, *p* =.476, indicating that at the highest rate onset rejection was completed in ten trials.

## Experiment 4

So far, we have tentatively explained the beneficial effect of a frequent, irrelevant visual onset on target discrimination as arising from its role as a warning signal, thereby enhancing participants’ preparedness to respond to the target (Posner & Boies, 1971). However, because the onset distractor consistently preceded the target by a fixed stimulus-onset asynchrony (SOA) of 100 ms, a slightly different explanation is also possible. One could argue that the onset provided reliable temporal information about the precise moment the target appeared, and that participants could have, in principle, exploited this information to improve task performance. Hence, given the peculiar temporal arrangement of the stimuli in Experiment 3, the observed facilitatory effect could stem from two similar but potentially different mechanisms: a generic “alerting” one whereby the abrupt visual onset triggers a brief boost in arousal that speeds up RTs (e.g., Posner & Cohen, 1984), and a more specific “temporal expectation” mechanism, dealing with the estimation of time of target occurrence based on the distractor presentation (Denison, 2024; Los & Agter, 2005).

To differentiate between these alternative explanations, we conducted a new experiment that was identical to Experiment 3, except that instead of using a single 100-ms SOA between the distractor and the target, we used two equiprobable SOAs: 100 ms and 500 ms. The inclusion of two distinct SOAs was intended to reduce the temporal predictability of the target relative to the distractor. If the facilitation observed in Experiment 3 with a single 100-ms SOA reflected a generic alerting phenomenon, the same effect should also be present in the current experiment despite the SOA variability. By contrast, if the facilitation depended on temporal expectation, such that participants used the distractor to anticipate the target occurrence, then the facilitatory effect should disappear or be substantially reduced at the 100-ms SOA due to the increased temporal uncertainty. However, the effect could still emerge at the longest 500-ms SOA, as participants could readily learn that if the target did not appear at the earlier 100-ms SOA, it would certainly appear at the later interval (Los & Agter, 2005).

### Method

#### Participants

We recruited 48 participants, who had a mean age of 26 years (*SD* = 7). Among them, 24 were female (i.e., 50% of the group). Data for this experiment were collected in 2025.

#### Stimuli and procedure

These were as in Experiment 3, but two equiprobable SOAs were used in onset-present trials: 100 ms or 500 ms.

#### Data analysis

The analytical plan and the software used for the statistical analyses were as in previous experiments. Error rate and RT differences were computed for the 100-ms and 500-ms SOA condition separately and analyzed using a within-participants ANOVA with the factors Block (1–4) and SOA (100 vs. 500 ms), and their interaction.

### Results and discussion

Of the total sample of 48 participants, two participants were excluded because their error rate was > 20%. Mean error rates are reported in Table 4. The ANOVA performed on error rate differences revealed no significant effect of the factor Block, *F*(2.91, 131.04) = 0.30, *p* =.818, *η*^*2*^*ₚ* <.01, SOA, *F*(1, 45) = 1.74, *p* =.194, *η*^*2*^*ₚ* =.037, or any Block × SOA interaction, *F*(2.64, 118.96) = 0.94, *p* =.413, *η*^*2*^*ₚ* =.021.

**Table 4:** Mean error rates and response times (RTs) by Block and Trial type for Experiment 4

**Block**	**Trial type**	**Error rate (SD)**	**RT (SD)**
1	Onset absent	4 (6)	499 (74)
1	Onset present 100-ms SOA	4 (4)	505 (83)
1	Onset present 500-ms SOA	5 (6)	493 (80)
2	Onset absent	4 (6)	497 (73)
2	Onset present 100-ms SOA	3 (3)	496 (72)
2	Onset present 500-ms SOA	4 (6)	487 (72)
3	Onset absent	5 (6)	491 (70)
3	Onset present 100-ms SOA	5 (4)	489 (64)
3	Onset present 500-ms SOA	4 (4)	496 (72)
4	Onset absent	5 (6)	488 (65)
4	Onset present 100-ms SOA	6 (8)	487 (68)
4	Onset present 500-ms SOA	6 (10)	478 (63)

Mean error rates and their SDs are reported as percentages. Mean RTs and their SDs are reported in milliseconds

**Fig. 3:**
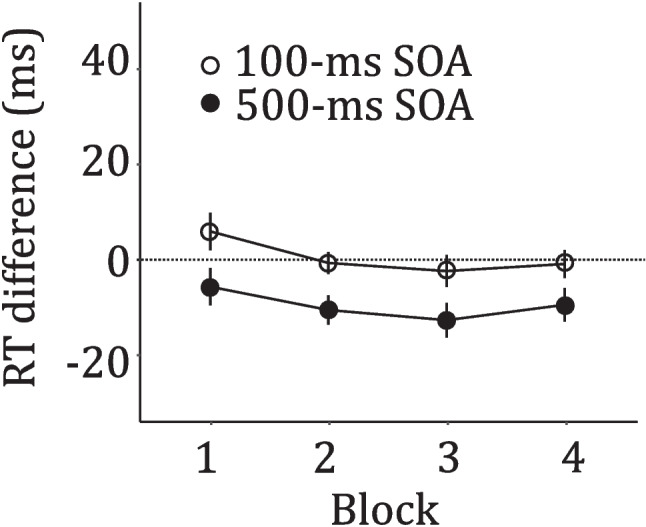
Effects of the onset presence on target discrimination as a function of block and stimulus-onset asynchrony (SOA). Mean response time (RT) difference (RT onset-present trials – RT onset-absent trials) by SOA (100 ms and 500 ms). Bars represent ± 1 standard error of the mean

The outlier-latency criterion resulted in the removal of 2.66% of the correct responses. Absolute mean RTs of correct responses in each condition are reported in Table 4. The ANOVA on the RT differences (Fig. 3, panel A) showed a significant main effect of SOA,* F*(1, 45) = 38.75, *p* <.001, *η*^*2*^*ₚ* =.463, while neither the main effect of Block, *F*(2.42, 108.90) = 1.18, *p* =.315, *η*^*2*^*ₚ* =.026, nor the Block × SOA interaction were significant, *F*(2.86, 128.73) = 0.26, *p* =.845, *η*^*2*^*ₚ* =.006. Post hoc comparisons showed that the distractor onset had a facilitatory effect at the 500-ms SOA condition only (100-ms SOA: *M* = 0.51, *SD* = 12.00; 500-ms SOA: *M* = −9.53, *SD* = 13.02; *t*(45) = −6.22, *p <*.001, *d* = −0.930).

The results were clear-cut, documenting that the facilitatory effect of the onset distractor was present only at the 500-ms SOA and disappeared completely at the 100-ms SOA, where it had been significant in Experiment 3. This pattern suggests that the onset distractor facilitation was primarily due to participants using the distractor to increase the temporal predictability of the target. In the current experiment, participants could not anticipate whether the target would have appeared 100 ms or 500 ms after the distractor. However, as time progressed within each trial, the probability of the target appearing at 500 ms increased, and by that point, its occurrence could be predicted with absolute certainty. This likely enabled participants to optimally orient their attention in time and/or to reduce the temporal uncertainty about the target (Los & Agter, 2005).

## General discussion

When human observers are engaged in a visual discrimination task, irrelevant sudden onsets appearing elsewhere in the visual field usually have a disrupting effect on performance, typically because attention is diverted from the target, thus slowing down its processing (Jonides & Yantis, 1988; Lamy & Egeth, 2003; Yantis & Jonides, 1990). Such a detrimental effect, however, diminishes as exposure to the visual onsets progresses (but see Ruthruff et al., 2019), a result that has been shown to be governed by habituation mechanisms (Kelley & Yantis, 2009; Turatto, 2023; Turatto, Bonetti, & Pascucci, 2018a, 2018b; Turatto, Bonetti, Pascucci, et al., 2018a, 2018b; Turatto et al., 2019; Turatto & Pascucci, 2016; Valsecchi & Turatto, 2025; Vatterott & Vecera, 2012; Won, 2021).

Although onset capture is subject to habituation, Turatto and Pascucci (2016) provided preliminary indications showing that when capture by a 50% rate onset was totally abolished on the first day of training, on the next two days participants became faster in discriminating the target in the presence of the irrelevant onset compared to its absence. In other words, while on the first day the irrelevant transients acted as distractors interfering with target discrimination, in the following days they facilitated target discrimination. This pattern of results suggests that irrelevant visual onsets could have a dual effect on the visual attention system, causing interference and facilitation, with the latter becoming manifest once habituation has abolished the former. Note that Turatto and Pascucci (2016) did not find any beneficial effect of onsets on target discrimination when the irrelevant transient occurred at a lower 20% rate (Experiment 1), which we attribute to the fact that at such a low onset rate habituation did not completely eliminate the distractor interference.

In the present study, we directly tested the hypothesis according to which onsets may have two concurrent and opposite effects by systematically manipulating the onset rate across three separate experiments. In support of our prediction, we found faster RTs in onset-present compared to onset-absent trials when the onset rate was sufficient to allow habituation mechanisms to eliminate capture, as observed in Experiment 2 (50% rate) and Experiment 3 (80% rate), but not in Experiment 1 (20% rate). Additionally, since the rate and strength of habituation of capture is proportional to the onset rate (Turatto & Pascucci, 2016; Turatto & Valsecchi, 2023), we expected the beneficial effect of onsets to emerge earlier the higher the onset rate. In line with this expectation, we found that capture was significant during the initial trials of block 1 in each experiment, but progressively diminished as a function of the onset rate, disappearing within the first ten trials in the 80% onset condition.^1^ Reciprocally, the onset benefit emerged progressively earlier as the onset rate increased. It was present in block 4 of Experiment 2 (50% onset rate) and in block 3 of Experiment 3 (80% onset rate). In other words, onset facilitation became manifest only after that capture vanished completely, and the faster habituation reached this level, the earlier the onset beneficial effect became visible.

Regarding the mechanism underlying the beneficial effects of an onset distractor presence on target processing, we should note that prior research with color-singleton distractors has already reported that, under certain conditions, the presence of an irrelevant feature singleton in the display can have a similar paradoxical beneficial effect on visual search. Indeed, target search can be faster in the presence of a color distractor in the display than in its absence (Gaspelin et al., 2015, 2017). This is because, in agreement with Gaspelin et al. (2025) and Sawaki and Luck (2013), the irrelevant salient distractor, which tends to trigger an unwanted attentional capture, would be suppressed and thus excluded from the searched items in the display, thus speeding up target selection (Gaspelin et al., 2015, 2017).

An alternative interpretation of the beneficial effect of onsets that we have reported refers to the notion of a “warning signal.” In particular, we note that in the present set of experiments, as in previous similar studies (e.g., Turatto, Bonetti, & Pascucci, 2018a, 2018b; Turatto, Bonetti, Pascucci, et al., 2018a, 2018b; Turatto et al., 2019; Turatto & Pascucci, 2016), the irrelevant visual onsets occurred just before the target. Hence, a possibility is that these events improved performance by acting as preparatory signals, optimizing the observer’s general readiness to process the subsequent target. In line with this possibility, Posner and Boies (1971) examined the effect of a warning signal on the subsequent imperative stimulus, and found that such signal can improve target processing. The authors argued that this effect arises because the anticipatory signal activates an alertness component of the attentional system, which is better prepared for delivering a faster response. The interval between the warning signal and the target would function as a “miniature vigilance situation,” where alertness is quickly developed and sustained over a short period to optimize target processing. Similarly, in our set of experiments onsets may have transiently increased participants’ alertness, thus favoring a preparatory state that facilitated a more efficient target processing. The possibility that a warning mechanism may improve task performance independently and, in our case despite the exogenous orienting of attention triggered by the peripheral onset, aligns with evidence showing the existence of three independent attentional networks, specifically devoted to alerting, orienting, and executive functions (Fan et al., 2002).

A similar but somewhat different explanation for the onsets facilitation could involve mechanisms of temporal orienting (or temporal uncertainty reduction), which can translate into the ability to flexibly and voluntarily orient attention to moments in time (Coull & Nobre, 1998; Denison, 2024; Los & Agter, 2005). A typical method to study temporal orienting is by manipulating the time interval between the onset of a warning cue and the target (i.e., the foreperiod). For example, when a constant foreperiod is used, participants are faster compared to when the foreperiod varies across trials (Correa et al., 2006; Los & Agter, 2005; Niemi & Näätänen, 1981). This suggests the presence of a mechanism that estimates the time between the warning signal or cue, and the imperative stimulus, allowing for top-down temporal preparation (Weinbach & Henik, 2012). When the time interval between the two stimuli is inconsistent or too long, temporal orienting is imprecise, and the facilitation effect is weakened. Given that in our paradigm the time interval between the onset distractor and the target was fixed, in principle we set the optimal conditions for temporal orienting to take place and speed up RTs. Temporal orienting could thus in principle explain our results because participants may have learned the time interval between the onset distractor and the target and exploited this reliable temporal information to better prepare to respond to the latter.

It has been debated as to whether alertness and temporal orienting are the same or represent different phenomena, and although there is evidence that the two share similar processes, they can also be dissociated (Weinbach & Henik, 2012). To this aim, in Experiment 4, we used two equiprobable SOAs, one identical to the SOA used in Experiment 3 (100 ms) and a longer one (500 ms) to reduce the temporal predictability of the target relative to the distractor. Under these conditions, the onset facilitatory effect was reliable at the longest 500-ms SOA but was absent at the shortest 100-ms SOA. This pattern of results indicates that temporal predictability of the target was a crucial determinant in the emergence of the facilitatory effect at the 100-ms SOA in Experiment 3, as the same SOA did not produce any facilitation under the unpredictable conditions of Experiment 4. By contrast, facilitation was still evident at the longest 500-ms SOA because the time of target occurrence was certain at this interval if the target had not appeared at the previous one (Los & Agter, 2005). It therefore seems safe to conclude that the onset facilitatory effect seen in Experiments 3 and 4 arose from temporal orienting or temporal uncertainty reduction with respect to the target appearance, rather than representing a generic alerting effect.

As a matter of fact, one might argue that the fixed onset–target interval may have been used not only to speed up RTs in the onset-present condition at higher onset rates (particularly at 80%), but also to reduce onset interference across blocks. In other words, the reduction in onset interference observed in our experiments would not result from a habituation (or from distractor suppression) mechanism, but rather from participants learning to anticipate target occurrence given the fixed onset–target delay. Naturally, the beneficial effect of such learning would emerge more quickly the higher the onset rate.

However, this alternative interpretation does not appear congruent with previous findings showing an attenuation of onset interference across blocks that is difficult, if not impossible, to attribute to a learned anticipation of the target occurrence. To begin with, Turatto and De Tommaso (2023), used a fixed onset–target delay of 150 ms throughout the experiment. The display-to-onset interval was also fixed (850 ms) during the first three blocks of trials (standard condition). In the fourth block, however, on 10% of the trials the display-to-onset interval was increased to 1,850 ms (delayed condition), while the onset–target delay remained unchanged (150 ms). This manipulation, which did not affect the participants’ ability to anticipate the target occurrence based on the irrelevant onset, dramatically increased capture in the delayed condition, whereas capture remained habituated in the standard condition. This pattern of results is incompatible with an interpretation of the reduced capture across blocks as a consequence of learned anticipation of the target occurrence based on the onset–target delay, which did not change.

Even more conclusive in dismissing the alternative interpretation are the results of a recent study by Burleson et al. (2025). The authors reported a clear reduction of peripheral onset interference on a central discrimination task in a paradigm where the onset–target delay varied randomly, trial by trial, between 50 ms and 200 ms (in 25-ms increments). This condition largely prevented participants from anticipating the target occurrence, except, perhaps, for the longest interval, but still onset capture progressively attenuated across blocks, as expected according to the habituation account.

A further consideration worth mentioning concerns the causes behind the distinctive attenuation-of-capture functions produced by the different onset rates. In the current paradigm, based on the distractor rate manipulation, we assume that capture attenuation was controlled by a habituation mechanism driven by distractor expectation, and in agreement with this, previous studies with onset distractors showed that habituation was stronger at higher onset rates (Turatto & Pascucci, 2016; Turatto & Valsecchi, 2023). In other words, similar to what was originally postulated for conditioning (Rescorla, 1988), habituation of capture, which is also based on a learning process, would be governed by a mechanism that tends to minimize the distractor prediction error. In the case of habituation of capture, the distractor prediction error would consist of the discrepancy between the actual distractor occurrence and how much the distractor was expected to occur (Turatto, 2023).

Alternatively, in agreement with the signal suppression account (Gaspelin et al., 2025; Luck et al., 2021), the attenuation of capture we documented could be explained by assuming that the onset distractor was suppressed as a function of how frequently it was encountered; that is, suppression increased with the distractor’s rate of occurrence. While there is considerable evidence in favor of the distractor suppression hypothesis (e.g., with evoked response potentials (ERPs), Sawaki & Luck, 2013; eye movements, Gaspelin et al., 2017; and single-unit studies in monkeys, Cosman et al., 2018; as reviewed by Gaspelin et al., 2025), which therefore remains a viable explanation for our findings, it should be noted that previous studies examining onset rejection have found no behavioral evidence that this is achieved through suppression, as indicated by the lack of target processing impairment at the onset distractor location (Valsecchi & Turatto, 2023, 2024).

Additionally, inter-trial facilitation by distractor position repetitions could also be responsible for the distinctive habituation-of-capture functions observed across onset-rate conditions, as distractor position repetitions were more likely in the 50% and 80% rate conditions compared to the 20% rate condition. Previous research with feature-singletons has shown that distractor position repetitions can reduce distractor interference compared to position switches (Geyer et al., 2008; Müller et al., 2009), independently of position distractor rate (Goschy et al., 2014). It is therefore possible that both distractor expectancy and position repetitions may have affected the capture attenuation functions in our study.

As for the nature of the capture we documented, we should note that our participants were prepared to attend to a target that appeared as an onset at the center of the screen. So, although onsets have been shown to capture attention irrespective of the attentional set used (Folk & Remington, 2015), it is very likely that capture in our study was in part modulated by top-down factors, as the distractor and the target were both onsets (Folk et al., 1992; Gibson & Kelsey, 1998). In addition, we cannot exclude that this contingent attentional capture component could have also favored an incidental learning of the temporal contingency between the onset distractor and the target, thus leading to the beneficial effect we have documented. However, irrespective of the specific contribution of any attentional set for onsets, here we have shown that onset capture is subject to different degrees of habituation depending on the onset rate, and that at high rates these visual events can have a positive effect on target processing.

In conclusion, irrelevant visual onsets may have two independent and, in some ways opposite, effects on visual performance: they can interfere with target identification because of the orienting response they tend to elicit, while also concurrently acting as signals that better prepare the observer to process the upcoming target. Yet, the beneficial effect of the latter process becomes evident only after habituation mechanisms, or other distractor suppressive mechanisms, have abolished the capture elicited by the same irrelevant visual onset.

## Data Availability

All data, program code, and other methods developed by others are cited in the text and listed in the references section. The experiment codes, data and analyses of the present study are available at: https://osf.io/7wevs/?view_only=ac86e08761654241abcfc49ffea0de5d

## Footnotes

1 The attentional capture decrement is not determined by the onset rate in absolute terms, but rather reflects a complex interaction of multiple cognitive mechanisms, which are influenced by various incidental factors associated with onset rate. Notable examples are the amount of practice with the irrelevant stimulation (Thompson & Spencer, 1966), the distractor expectation (Turatto & Valsecchi, 2023), and the inter-trial priming (Müller et al., 2009), but others may be involved.
